# Association of step counts over time with the risk of chronic disease in the *All of Us* Research Program

**DOI:** 10.1038/s41591-022-02012-w

**Published:** 2022-10-10

**Authors:** Hiral Master, Jeffrey Annis, Shi Huang, Joshua A. Beckman, Francis Ratsimbazafy, Kayla Marginean, Robert Carroll, Karthik Natarajan, Frank E. Harrell, Dan M. Roden, Paul Harris, Evan L. Brittain

**Affiliations:** 1 Vanderbilt Institute of Clinical and Translational Research, Vanderbilt University Medical Center, Nashville, TN USA; 2 Department of Biostatistics, Vanderbilt University School of Medicine, Nashville, TN USA; 3 Division of Cardiovascular Medicine, Vanderbilt University Medical Center, Nashville, TN USA; 4 Department of Biomedical Informatics, Vanderbilt University Medical Center, Nashville, TN USA; 5 Department Biomedical Informatics, Columbia University, New York, NY USA; 6 Department of Medicine and Biomedical Informatics, Vanderbilt University Medical Center, Nashville, TN USA; 7 Department of Biomedical Informatics, Biomedical Engineering and Biostatistics, Vanderbilt University Medical Center, Nashville, TN USA

**Keywords:** Risk factors, Preventive medicine

## Abstract

The association between physical activity and human disease has not been examined using commercial devices linked to electronic health records. Using the electronic health records data from the *All of Us* Research Program, we show that step count volumes as captured by participants’ own Fitbit devices were associated with risk of chronic disease across the entire human phenome. Of the 6,042 participants included in the study, 73% were female, 84% were white and 71% had a college degree, and participants had a median age of 56.7 (interquartile range 41.5–67.6) years and body mass index of 28.1 (24.3–32.9) kg m^–2^. Participants walked a median of 7,731.3 (5,866.8–9,826.8) steps per day over the median activity monitoring period of 4.0 (2.2–5.6) years with a total of 5.9 million person-days of monitoring. The relationship between steps per day and incident disease was inverse and linear for obesity (*n* = 368), sleep apnea (*n* = 348), gastroesophageal reflux disease (*n* = 432) and major depressive disorder (*n* = 467), with values above 8,200 daily steps associated with protection from incident disease. The relationships with incident diabetes (*n* = 156) and hypertension (*n* = 482) were nonlinear with no further risk reduction above 8,000–9,000 steps. Although validation in a more diverse sample is needed, these findings provide a real-world evidence-base for clinical guidance regarding activity levels that are necessary to reduce disease risk.

## Abstract (web summary)

Step count monitoring with Fitbit devices in the *All of Us* Research Program enabled quantification of the benefits of physical activity across a wide range of chronic diseases over the course of an extended time frame—a median of 4 years.

## Main

Physical activity can be quantified and tracked by wearables that are used widely by the public. Prior studies consistently show that taking fewer steps per day^1–6^ is associated with higher risk of all-cause mortality and cardiovascular disease. These studies raise public awareness of the importance of engaging in physical activity, but study design limitations also leave important questions unanswered. First, prior studies assessed physical activity either by self-reported questionnaires or by having participants wear a research-grade device for a brief monitoring period (most often 7 days)^1–4^. As a result, activity may be under- or over-reported. Moreover, no information is reported about activity levels between the baseline period and when outcomes are assessed at follow-up, often many years later. Second, prior studies have focused on a relatively targeted set of outcomes limited to mortality, diabetes and cardiovascular disease. Little is known about the impact of activity over time on developing chronic diseases across the full human phenome, which represents the sum of human traits and conditions resulting from genetic and behavioral variation in a population^7^.

The *All of Us* Research Program (AoURP) is an initiative that is accumulating multiple streams of health-related information (for example, electronic health records (EHRs), genomics, physical measures, participant surveys and wearables such as Fitbit) in 1,000,000 or more Americans and includes a focus on populations usually under-represented in biomedical research to date^8^. The rich EHR data within AoURP can be used to identify the incidence of chronic conditions across the human phenome^9^. Thus, the AoURP dataset provides a unique opportunity to directly examine the effects of physical activity over time on health outcomes using wearables and clinical data.

The purpose of this study was to examine the associations between physical activity over time and incident chronic diseases. Based on previous literature^1,3^, we hypothesized that more steps per day over time will be associated with lower incidence of chronic diseases. We also sought to identify empiric, evidence-based activity levels associated with risk of, and protection from, chronic disease, which could inform public health guidance on physical activity.

## Results

### Participant characteristics

**Extended Data Fig. 1:**
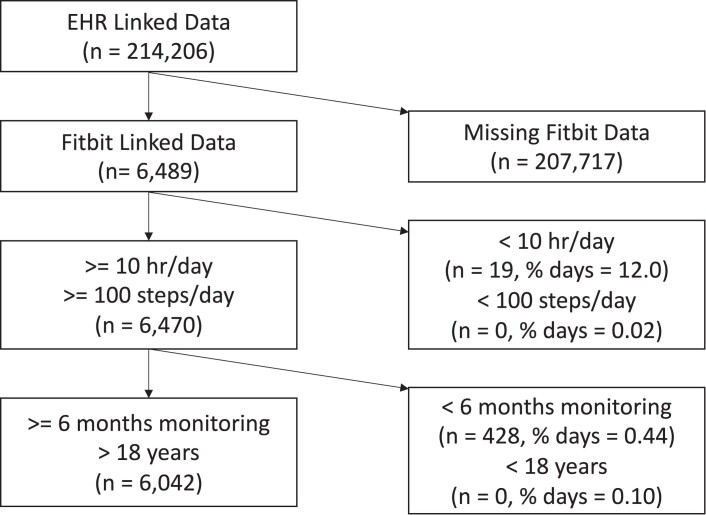
Consort diagram showing inclusion/exclusion criteria. The flow diagram graphically depicts the steps that were utilized to derive the analytical sample that met the inclusion and exclusion criteria. EHR, electronic health records.

Of the 329,070 AoURP participants at the time of our analysis, 214,206 participants had consented to share EHR data. Of those sharing EHR data, 6,042 participants linked their own Fitbit device, had valid Fitbit data over at least 6 months of total monitoring and were aged at least 18 years at any time during the monitoring period (Extended Data Fig. 1). Only 0.02% and 0.44% of total days were excluded given they had fewer than 100 steps and <6 months of monitoring, respectively. Participants had a median age of 56.7 years (interquartile range (IQR) 41.5–67.6) and median body mass index (BMI) of 28.1 kg m^–2^ (IQR 24.3–32.9) at baseline (Table 1). Nearly 73%, 84% and 71% were female, white and with a college degree, respectively. The median daily step counts were 7,731 (IQR 5,867–9,827) steps per day over Fitbit monitoring duration of 4.0 years (IQR 2.2–5.6), representing 5,991,662 person-days of monitoring and nearly 50.6 billion total steps. Participants with Fitbit and EHR data were more likely to be white, female and to have a lower burden of medical comorbidities compared with those sharing EHR data alone (Table 1).

**Table 1:** Participant baseline characteristics for those included versus excluded from the analytical cohort

	Included	Excluded	*P* value
Variable	Median (IQR) or *N* (%)		
Subjects (*n*)	6,042	208,164	
Age	56.69 (41.45–67.62)	56.91 (40.96–68.24)	0.373
Race			<0.001
Black	336 (5.6)	45,661 (21.9)	
Other	309 (5.1)	11,112 (5.3)	
White	5,072 (83.9)	108,141 (51.9)	
Sex at birth			<0.001
Female	4,379 (72.5)	126,159 (60.6)	
Male	1,579 (26.1)	77,969 (37.5)	
Ethnicity			<0.001
Hispanic or Latino	376 (6.2)	41,638 (20.0)	
Not Hispanic or Latino	5,590 (92.5)	160,368 (77.0)	
Education			<0.001
College degree	4,317 (71.4)	82,407 (39.6)	
Some college	1,346 (22.3)	53,973 (25.9)	
No college	356 (5.9)	66,925 (32.2)	
BMI	28.10 (24.32–32.85)	28.80 (24.70–34.10)	<0.001
Baseline conditions			
CAD	170 (2.8)	14,684 (7.1)	<0.001
Cancer	1,429 (23.7)	58,050 (27.9)	<0.001
Smoking (100 cigarettes)			
>100 cigarettes	1,932 (32.0)	84,466 (40.6)	<0.001
Alcohol			
≥1 drink	5,846 (96.8)	177,735 (85.4)	<0.001
Fitbit variables			
Duration (years)	3.99 (2.15–5.58)		
Average daily steps	7,731.30 (5,866.84–9,826.85)		

Data are median (IQR) for continuous variables and frequency (percentage) for categorical variables.

Participants were excluded because of not having EHR and Fitbit data or valid Fitbit data for at least 6 months. For included participants, missingness for each variable was as follows: Race, 325 (5.4%); Sex, 84 (1.4%); Ethnicity, 76 (1.3%); BMI, 2,644 (43.8%); Smoking, 108 (1.8%); Education, 23 (0.4%); Alcohol, 31 (0.5%). For excluded participants, missingness for each variable was as follows: Race, 43,250 (20.8%); Sex, 4,036 (1.9%); Ethnicity, 6,158 (3.0%); BMI, 51,496 (24.7%); Smoking, 6,094 (2.9%); Education, 4,859 (2.3%); Alcohol, 6,477 (3.1%). For included participants, BMI had a high amount of missingness because the measurement must have occurred before the Fitbit monitoring period. Mann–Whitney *U* and chi-squared tests for continuous and categorical variables, respectively, were used to compare these clinical characteristics for the participants who were excluded versus included in the analytical dataset for this study.

### Daily steps and chronic diseases across human phenome

**Fig. 1:**
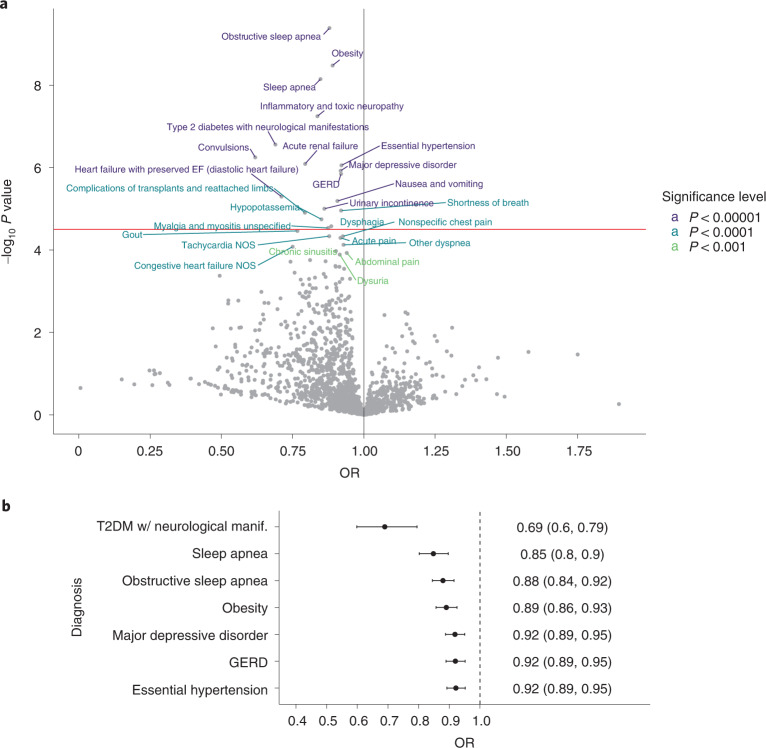
Hypothesis-generating analysis to explore relation between daily steps and prevalent chronic disease across human phenome. **a**, Negative log base-10 *P* values for each phecode are plotted as a function of the OR from the corresponding logistic regression with average daily step count. EF, ejection fraction; NOS, not otherwise specified. OR is reported per 1,000 step count increase, as adjusted for age, race and sex. All phecodes occurred after 6 months of Fitbit monitoring and not before. Horizontal red line indicates the Bonferroni corrected α level of 3.1856 × 10^–5^, accounting for all phecodes used. Vertical line is OR = 1. **b**, OR and 95% CI to quantify the association of increasing daily step counts with selected outcomes, that is type 2 diabetes mellitus (T2DM) with (w/) neurological manifestation (manif.) (sample size, *N* = 5,976), sleep apnea (*N* = 5,699), obstructive sleep apnea (*N* = 5,518), obesity (*N* = 5,267), major depressive disorder (*N* = 5,370), GERD (*N* = 5,091) and essential hypertension (*N* = 4,897). The points represent OR and error bars represent 95% CI. The values toward the right of the figure represent OR (95% CI) values in text format. All models were adjusted for age, race and sex.

Our first analysis involved exploratory examination of the relationship between average step counts over an individual’s entire monitoring period and incident disease across all 1,711 phecodes. Figure 1a,b shows these data in a logistic regression model adjusted for age, sex and race set to an effect size (odds ratio (OR)) per 1,000 step increase. ORs <1 indicate that higher step counts were associated with lower risk of each condition. Incident chronic conditions with the largest effect sizes that met the adjusted statistical significance threshold across the human phenome after a minimum of 6 months of monitoring were obstructive sleep apnea (*n*/*N* = 342/5,518, OR (95% confidence intervals (CI)) = 0.88 (0.84, 0.92)), obesity (*n*/*N* = 380/5,267, OR (95% CI) = 0.89 (0.86, 0.93)), type 2 diabetes with neurological manifestations (*n*/*N* = 37/5,976, OR (95% CI) = 0.69 (0.60, 0.79)), hypertension (*n*/*N* = 498/4,897, OR (95% CI) = 0.92 (0.89, 0.95)), gastroesophageal reflux disease (GERD) (*n*/*N* = 451/5,091, OR (95% CI)= 0.92 (0.89, 0.95)) and major depressive disorder (MDD) (*n*/*N* = 483/5,370, OR (95% CI) = 0.92 (0.89, 0.95)) (Fig. 1 and Supplementary Table 2).

**Extended Data Fig. 2:**
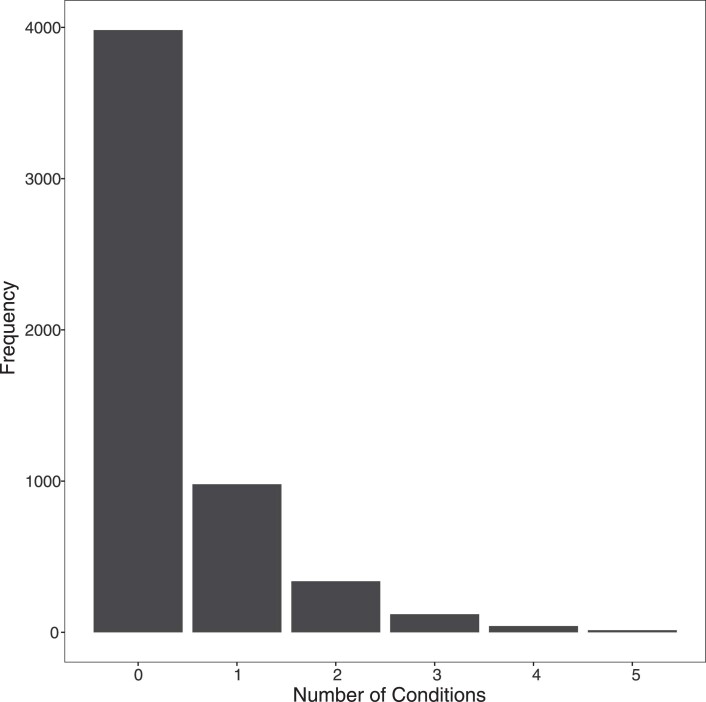
Frequency of multiple conditions by number. The bar chart depicts number of participants who reported having multimorbidity among 6 conditions, i.e. diabetes, hypertension, gastroesophageal reflux disease, major depressive disorder, obesity and sleep apnea.

We focused subsequent analyses on chronic conditions with a plausible biological link to activity levels including diabetes, hypertension, GERD, MDD, obesity and sleep apnea (Supplementary Table 1). Type 2 diabetes codes with neurological manifestation were combined with codes for type 2 diabetes, and sleep apnea and obstructive sleep apnea were combined into a single condition given their phenotypic and diagnostic overlap. The conditions that were of interest in time to event analyses often coexist clinically, but multimorbidity among these six conditions was rare in this cohort (Extended Data Fig. 2). In addition to removing conditions that did not meet the statistical significance threshold in logistic regression, acute conditions (acute renal failure), nonspecific diagnoses (nausea and vomiting, shortness of breath, urinary incontinence, dysphagia, complications of transplants, inflammatory and toxic neuropathy), those with few events, that is, *n* ≤ 50 (convulsions, heart failure with preserved ejection fraction) and those with little to no plausible link to activity (hypopotassemia) were not pursued in subsequent analyses.

### Time-varying analysis of daily step counts and disease risk

**Fig. 2:**
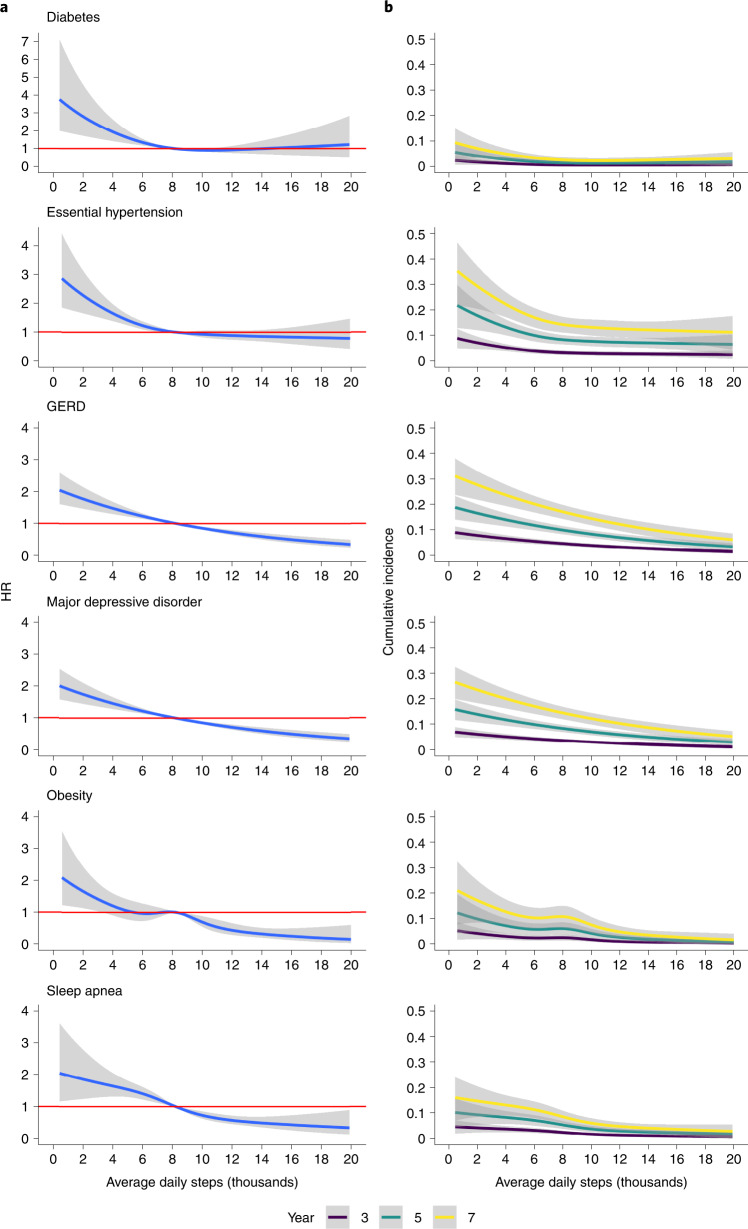
Relation between daily steps over time and incident chronic disease. **a**, Cox models were used to compute HRs as a function of average daily step count. Median step counts of 8,160 (diabetes), 8,290 (essential hypertension), 8,260 (GERD), 8,210 (major depressive disorder), 8,280 (obesity) and 8,220 (sleep apnea) were used as reference. **b**, Cumulative incidence by year for each outcome as a function of average daily step count. Shaded area represents 95% CI. All the Cox models were adjusted for age, race, sex, CAD, cancer, BMI, systolic blood pressure, education level, smoking and alcohol use.

In the Cox models, 15.4–16.0% of months (that is, 4.7–4.9% of days) were excluded due to fewer than 15 valid days of data. Figure 2a shows the relationship between step counts and adjusted hazard ratio (HR) for an incident diagnosis of each condition referenced to the median daily average steps for the entire cohort. The median values ranged from 8,160 to 8,290 steps per day across the different analytical cohorts created to investigate each incident diagnosis.

**Extended Data Fig. 3:**
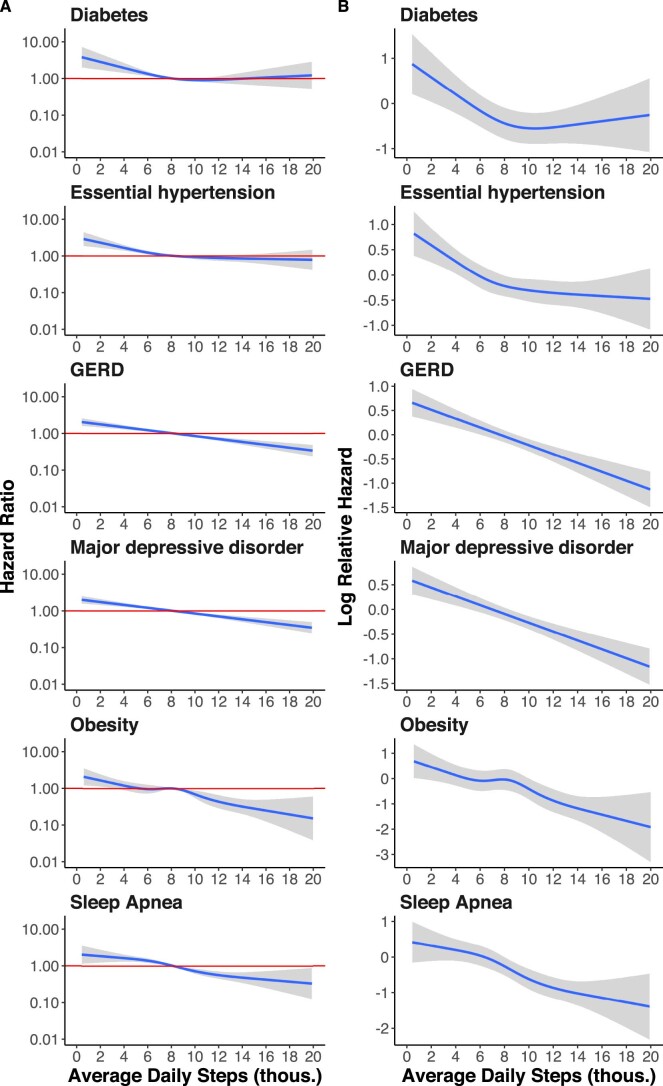
Relation between daily steps counts and incident risk of chronic diseases. Relation between daily steps counts and incident risk of chronic diseases. **a**: Adjusted Hazard ratio on log-transformed scale as a function average daily steps for each select outcome. **b**: Log relative hazard as a function of average steps for each selected outcome. All the models were adjusted for age, race, sex, coronary artery disease, cancer, body mass index, systolic blood pressure, education level, smoking, and alcohol use. Shaded area represents 95%confidence interval.

The relationship between steps and incident disease was inverse (all *P* < 0.001) and linear for obesity, sleep apnea, GERD and MDD (chunk tests for nonlinearity were nonsignificant for those conditions) with a lower risk of each diagnosis at higher step counts. For example, in comparison with the median step count, the risk of obesity declined by 31% (HR 0.69, 95% CI 0.53, 0.88) when steps were increased to 10,000 per day. In contrast, incident diabetes and hypertension had a nonlinear relationship (chunk tests for nonlinearity *P* < 0.05 for both) with step counts demonstrating inflections points at approximately 9,000 and 8,000 steps, respectively, where risk plateaued at higher step counts. Figure 2b shows the estimated cumulative rates of a new diagnosis for 3, 5 and 7 years, which shows how risk changes when an average step count is maintained over time. In general, incident risk for all the years is higher at lower step counts and increases over time when a given step count is maintained. For example, the risk of new hypertension diagnosis at 6,000 steps per day is maintained at 4%, 10% and 17% at 3, 5 and 7 years, respectively. Risk for all conditions except hypertension asymptotically approached zero at very high daily step counts. Extended Data Fig. 3a shows the HR on the log-transformed scale and Extended Data Fig. 3b shows the relationships between incident disease and step counts as a log relative hazard function that is not indexed to the median step count value. These results confirm general relationships between higher daily steps and lower disease risk. Table 2 models the adjusted HR for each condition when moving from the 25th percentile for average daily step counts to the 75th percentile, which gives insight into the distribution of risk across the spectrum of step counts. Participants with step counts at the 75th percentile have 24–52% lower risk of developing diabetes, hypertension, GERD, MDD, obesity and sleep apnea, compared with those who were in 25th percentile, adjusted for a priori covariates. Results were similar across limited and full a priori models as well as those that accounted for wear time and baseline step counts over the first 3 and 6 months (Table 2, Models 1–5).

**Table 2:** HRs, 95% CI and *P* values for 75th step count percentile versus 25th percentile with respect to each continuous Cox model and diagnosis

Model/diagnosis	Sample size (*N*)	Events (*n*)	25th percentile (thousands)	75th percentile (thousands)	HR (75% versus 25%)	95% CI	*P* value
**Model 1**							
Diabetes	5,124	156	6.05	10.63	0.44	0.28, 0.68	<0.001
Hypertension	4,437	482	6.18	10.73	0.71	0.55, 0.91	0.007
GERD	4,613	432	6.14	10.76	0.64	0.56, 0.73	<0.001
MDD	4,884	467	6.09	10.72	0.63	0.55, 0.72	<0.001
Obesity	4,774	368	6.16	10.77	0.52	0.43, 0.62	<0.001
Sleep apnea	4,922	348	6.11	10.70	0.53	0.45, 0.63	<0.001
**Model 2**							
Diabetes	5,124	156	6.05	10.63	0.68	0.56, 0.84	<0.001
Hypertension	4,437	482	6.18	10.73	0.76	0.59, 0.98	0.033
GERD	4,613	432	6.14	10.76	0.66	0.57, 0.76	<0.001
MDD	4,884	467	6.09	10.72	0.66	0.57, 0.76	<0.001
Obesity	4,774	368	6.16	10.77	0.59	0.43, 0.82	0.001
Sleep apnea	4,922	348	6.11	10.70	0.48	0.35, 0.65	<0.001
**Model 3**							
Diabetes	5,124	156	6.05	10.63	0.67	0.54, 0.83	<0.001
Hypertension	4,437	482	6.18	10.73	0.75	0.58, 0.98	0.031
GERD	4,613	432	6.14	10.76	0.64	0.55, 0.74	<0.001
MDD	4,884	467	6.09	10.72	0.67	0.59, 0.78	<0.001
Obesity	4,774	368	6.16	10.77	0.59	0.42, 0.81	0.001
Sleep apnea	4,922	348	6.11	10.70	0.54	0.45, 0.64	<0.001
**Model 4**							
Diabetes	5,124	156	6.05	10.63	0.77	0.69, 1.0	<0.05
Hypertension	4,437	482	6.18	10.73	0.81	0.61, 1.07	0.14
GERD	4,613	432	6.14	10.76	0.71	0.59, 0.84	<0.001
MDD	4,884	467	6.09	10.72	0.69	0.58, 0.82	<0.001
Obesity	4,774	368	6.16	10.77	0.56	0.40, 0.80	0.001
Sleep apnea	4,922	348	6.11	10.70	0.49	0.35, 0.68	<0.001
**Model 5**							
Diabetes	5,124	156	6.05	10.63	0.78	0.59, 1.02	0.07
Hypertension	4,437	482	6.18	10.73	0.82	0.62, 1.08	0.158
GERD	4,613	432	6.14	10.76	0.71	0.59, 0.86	<0.001
MDD	4,884	467	6.09	10.72	0.70	0.59, 0.84	<0.001
Obesity	4,774	368	6.16	10.77	0.56	0.40, 0.79	0.001
Sleep apnea	4,922	348	6.11	10.70	0.49	0.35, 0.68	<0.001

Model 1 included steps (time-varying), age, race and sex. Model 2 = Model 1 plus systolic blood pressure, CAD, cancer, smoking, education, alcohol and body mass index. Model 3 = Model 2 plus Fitbit wear time (time-varying). Model 4 = Model 2 plus baseline step counts (averaged over first 3 months). Model 5 = Model 2 plus baseline step counts (averaged over first 6 months).

**Fig. 3:**
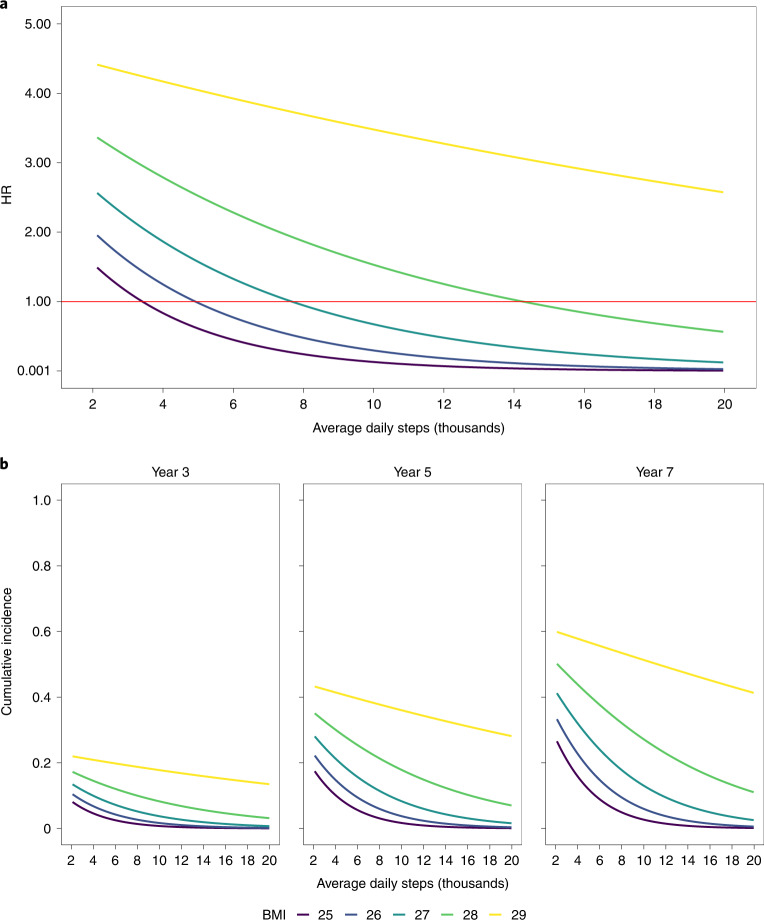
Relation between daily step counts and incident risk of obesity. **a**, Cox models were used to compute HR for obesity (outcome) as a function of average daily step count as stratified by BMI of 25–29 kg m^–2^. A median step count of 8,594 steps was used as reference. **b**, Cumulative incidence by year as a function of average daily step count and as stratified by BMI of 25–29 kg m^–2^. The model is identical to models previously described except BMI was allowed to interact linearly with the average daily step count.

We next modeled risk of obesity as a function of baseline BMI, daily step counts and BMI*steps interaction using recorded BMI values rather than diagnostic codes. We included only individuals who never had a recorded BMI > 30 kg m^–2^ or a coded diagnosis of obesity at any time before or during the first 6 months of monitoring (*N* = 1,067). This analysis was designed to address potential concerns regarding incidence estimates confounded by conditions that were prevalent but undiagnosed at baseline. Figure 3 and Supplementary Table 3 show that the risk of obesity decreases substantially at higher step counts, even when the baseline BMI is near the threshold for obesity. For example, when starting at a baseline BMI of 28 kg m^–2^, risk of obesity is reduced by 36% (95% CI 20–49%) when step counts increase from the 25th to 75th percentile. This increase in step counts resulted in a 50% reduction in cumulative incidence of obesity at 5 years from 16% (95% CI 11, 20) at the 25th percentile compared with 8% (95% CI 5, 12) at the 75th percentile.

**Extended Data Fig. 4:**
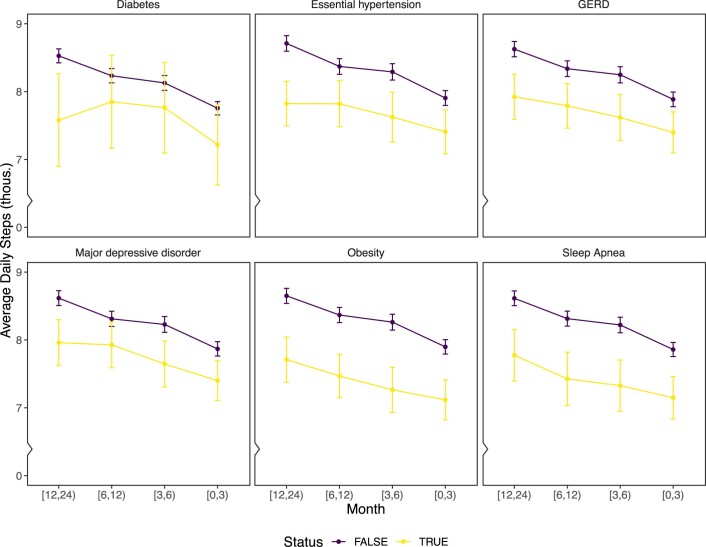
Trajectory of average daily step counts (mean and 95% confidence interval) among participants who did (status = True) and did not (status = False) develop each of the conditions, that is, diabetes (Sample Size (N) = 5124), essential hypertension (N = 4437), GERD (N = 4613), major depressive disorders (N = 4884), obesity (N = 4774) and sleep apnea (N = 4922) that were examined in Cox Models. Trajectory of average daily step counts (mean and 95% confidence interval) among participants who did (status = True) and did not (status = False) develop each of the conditions, that is, diabetes (Sample Size (N) = 5124), essential hypertension (N = 4437), GERD (N = 4613), major depressive disorders (N = 4884), obesity (N = 4774) and sleep apnea (N = 4922) that were examined in Cox Models. Indentation (>) represents the break in y-axis.

**Extended Data Fig. 5:**
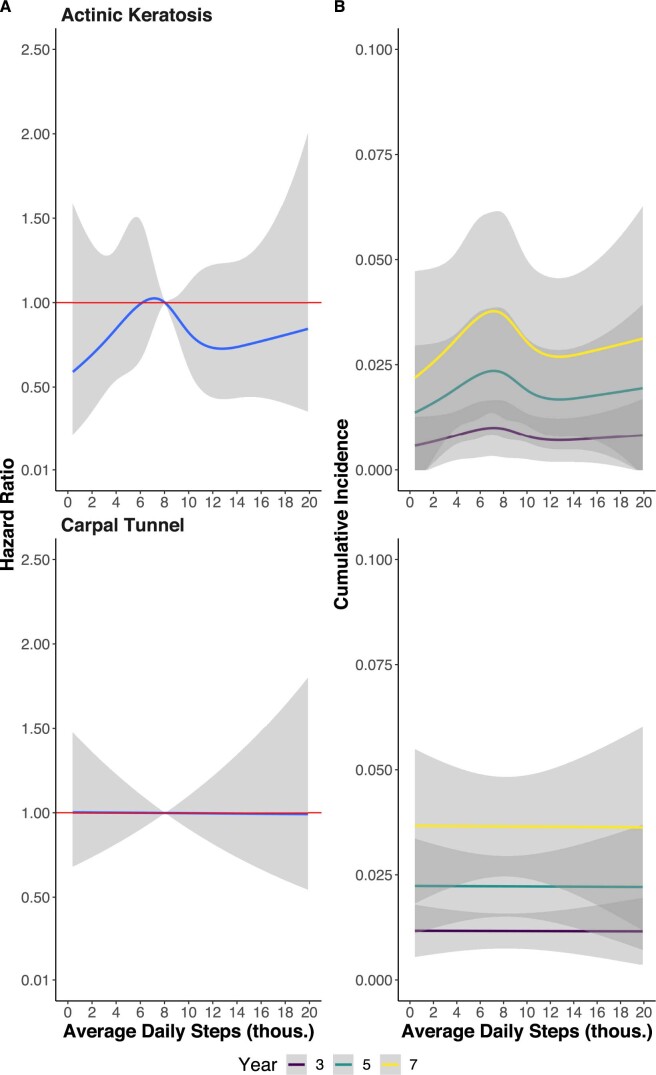
Relation between daily step counts and incident risk of actinic keratosis and carpal tunnel syndrome. Relation between daily step counts and incident risk of actinic keratosis and carpal tunnel syndrome. **a**: Adjusted Hazard ratios as a function of average daily step count. All the models were adjusted for age, race, sex, coronary artery disease, cancer, body mass index, systolic blood pressure, education level, smoking, and alcohol use. Reference for steps was the median steps count at 8,064 steps. **b**: Cumulative incidence by year as a function of average daily step count for each selected outcome. Shaded area represents 95%confidence interval.

A trajectory analysis in which steps were plotted at discreet time periods before disease diagnosis showed lower baseline step counts and a prediagnosis plateau (particularly for hypertension and depression) among those with incidence disease (Extended Data Fig. 4). Based on the findings of Models 4 and 5 shown in Table 2, accounting for baseline daily steps averaged over the first 3 or 6 months in the separate Cox models, in addition to a priori covariates, did not change the relation between daily steps over time with incident conditions. We performed a falsification analysis to examine the association between step counts and incident diagnoses with no expected relationship to step counts. As expected, we found no association between daily step counts and risk of carpal tunnel syndrome (*n*/*N* = 131/5,269) or actinic keratosis (*n*/*N* = 167/5,242 incident diagnoses) (Extended Data Fig. 5).

### Daily step counts, intensity and incident chronic disease

**Extended Data Fig. 6:**
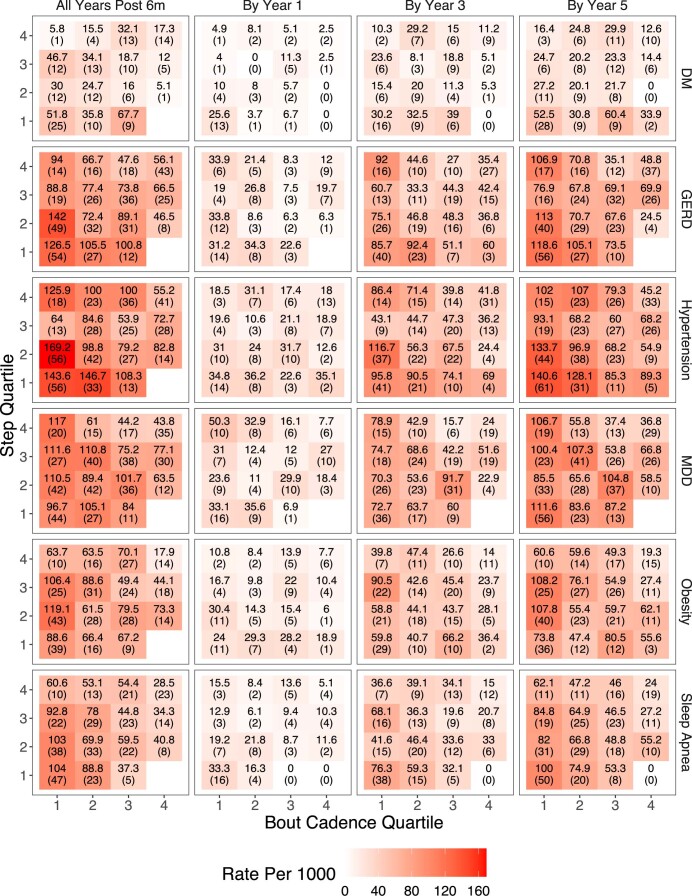
Incidence rates (number of events shown in brackets) for each diagnosis as a function of step counts and bout cadence (step intensity) quartiles. Incidence rates (number of events shown in brackets) for each diagnosis as a function of step counts and bout cadence (step intensity) quartiles. Incidence rates is number of cases per 1000 people for each diagnosis as a function of step and bout cadence quartiles. Bout cadence referred to steps per minute computed by averaging the steps over the time when participant engaged in ≥2 consecutive minutes at ≥60 steps/minute across all the valid days. Step quartile intervals (thous.) are as follows: 1: [549,5.87]; 2: [5.87,7.73]; 3: [7.73,9.83]; 4: [9.83,33.6]. Bout cadence quartiles are as follows: 1: [68.1,83.6]; 2: [83.6,88.7]; 3: [88.7,94.3]; 4: [94.3,158].

**Extended Data Fig. 7:**
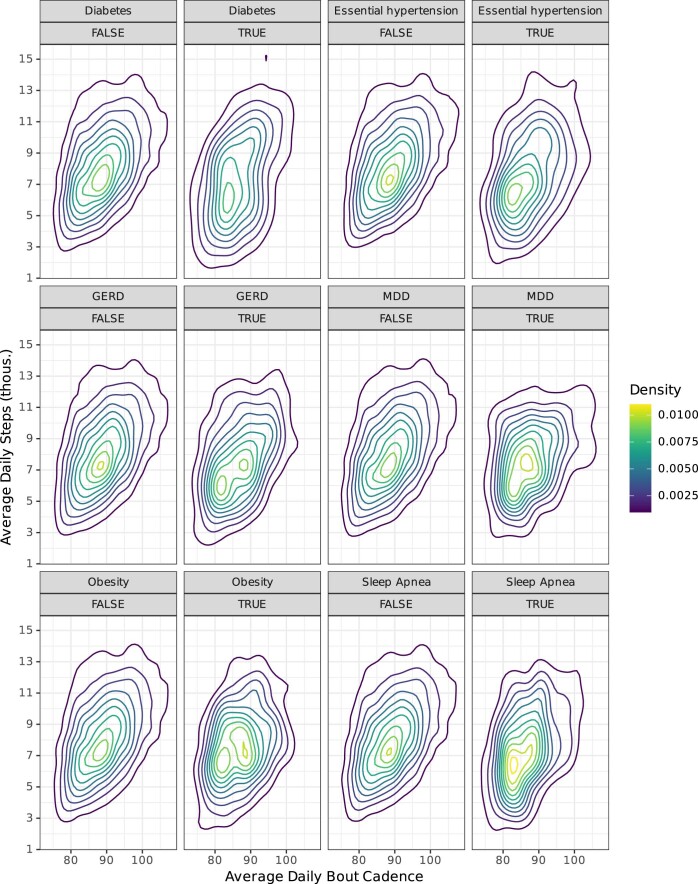
Probability density plot examining the relation between step counts and bout cadence (step intensity) on continuous spectrum in participants who developed (represented as TRUE) vs. did not develop the condition (represented as FALSE). Probability density plot examining the relation between step counts and bout cadence (step intensity) on continuous spectrum in participants who developed (represented as TRUE) vs. did not develop the condition (represented as FALSE). Bout cadence referred to steps per minute computed by averaging the steps over the time when participant engaged in ≥2 consecutive minutes at ≥60 steps/minute across all the valid days.

**Extended Data Fig. 8:**
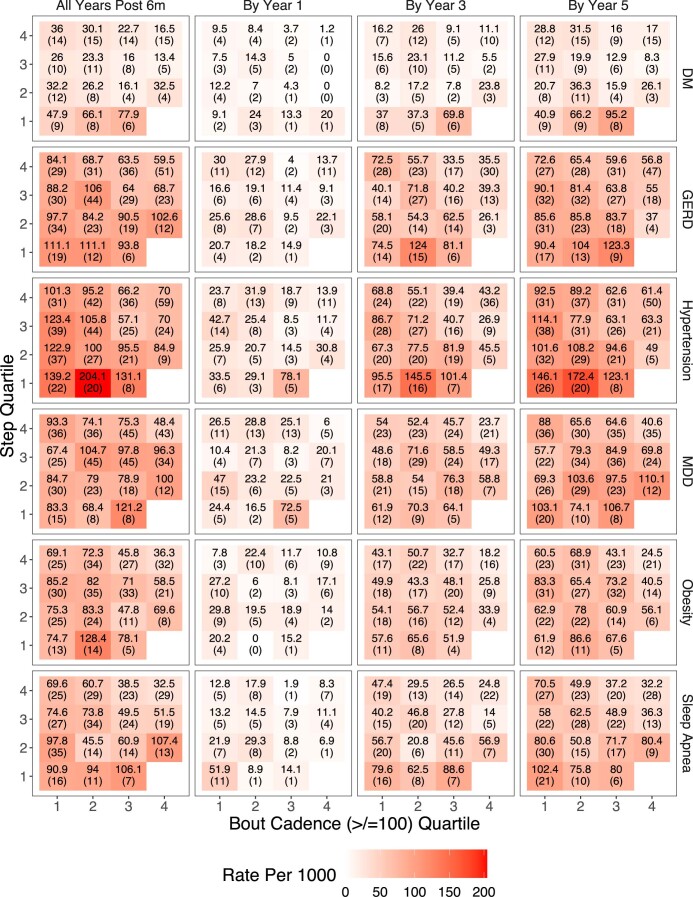
Incidence rates (number of events shown in brackets) for each diagnosis as a function of step counts and bout cadence (step intensity) quartiles. Incidence rates (number of events shown in brackets) for each diagnosis as a function of step counts and bout cadence (step intensity) quartiles. Incidence rates is number of cases per 1000 people for each diagnosis as a function of step and bout cadence quartiles. Bout cadence referred to steps per minute computed by averaging the steps over the time when participant engaged in ≥2 consecutive minutes at ≥100 steps/minute across all the valid days. Step quartile intervals (thous.) are as follows: 1: [549,5.87]; 2: [5.87,7.73]; 3: [7.73,9.83]; 4: [9.83,33.6]. Bout cadence quartiles are as follows: 1: [102,109]; 2: [109,111]; 3: [111,114]; 4: [113,207].

**Extended Data Fig. 9:**
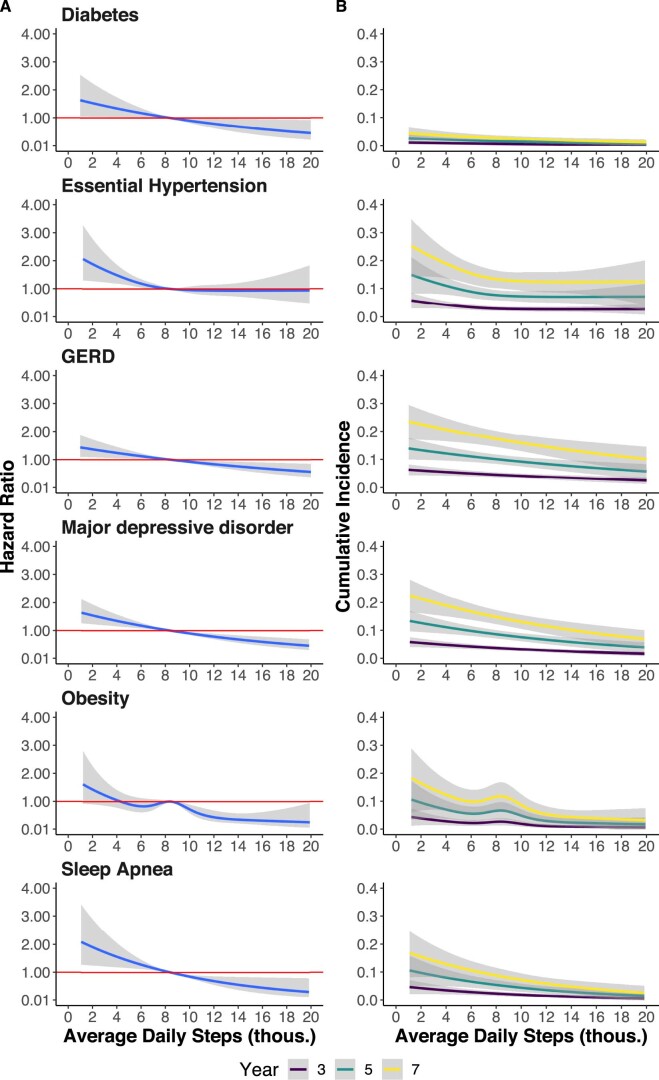
Relation between daily steps counts and incident risk of chronic diseases. Relation between daily steps counts and incident risk of chronic diseases. A: Hazard ratio as a function of average daily steps in thousands for each outcome. B: Cumulative incidence as a function of average daily steps for each selected outcome by year. Results of Cox model with average daily bout cadence (step intensity) as covariate, in addition to other covariates: age, race, sex, coronary artery disease, cancer, body mass index, systolic blood pressure, education level, smoking, and alcohol use. Bout cadence referred to steps per minute computed by averaging the steps over the time when participant engaged in ≥2 consecutive minutes at ≥60 steps/minute across all the valid days. Shaded area represents 95%confidence interval.

Daily step counts and intensity (defined using a steps per minute threshold that indicates slow walking) were positively correlated (ρ coefficient ranges from 0.48 to 0.87, *P* < 0.001). We observed a gradient of higher disease risk at the intersections of lower daily step counts and lower bout cadence quartiles compared with higher daily step counts and higher bout cadence quartiles (Extended Data Fig. 6). We saw similar trends when this relation was examined on a continuous basis using a probability density plot (Extended Data Fig. 7). When step intensity was defined using the moderate to vigorous intensity steps per minute threshold, similar findings were observed albeit with lower rates of incident disease (Extended Data Fig. 8). Daily step counts remained significantly associated with each condition (all chunk tests *P* < 0.05) after accounting for step intensity (Extended Data Fig. 9 and Supplementary Table 4). Specifically, the effect estimates, that is, HR, for step counts for diabetes, obesity, sleep apnea, GERD and MDD ranged from 0.64 to 0.81 (Supplementary Table 4).

**Extended Data Fig. 10:**
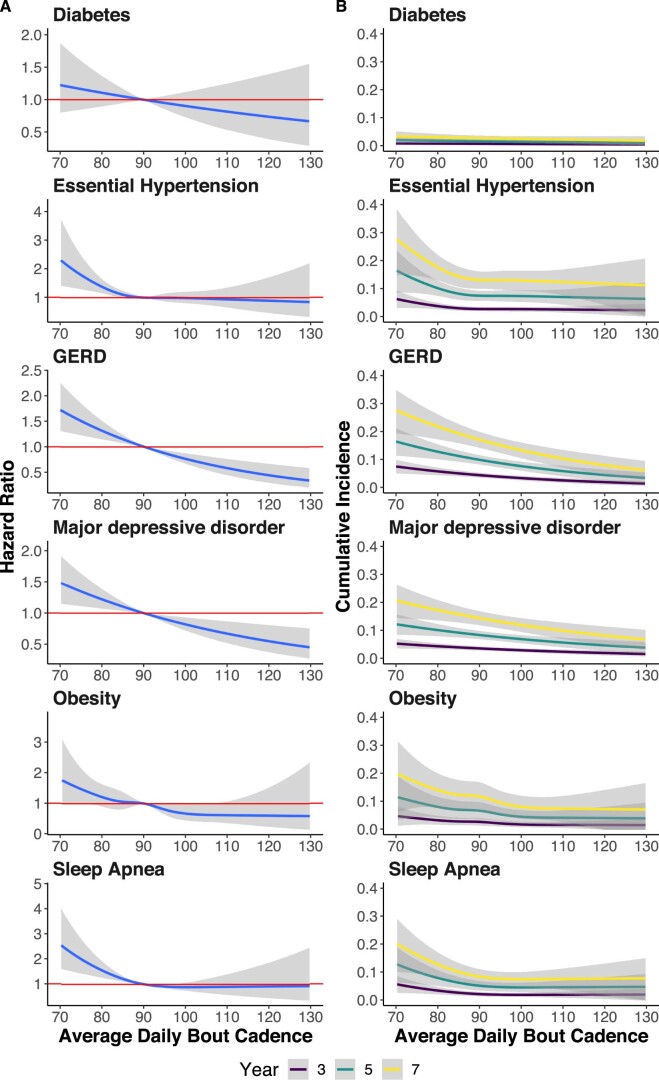
Relation between daily bout cadence (step intensity) and incident risk of chronic diseases. Relation between daily bout cadence (step intensity) and incident risk of chronic diseases. **a**: Hazard ratio as a function of average daily bout cadence (step intensity) for each outcome. **b**: Cumulative incidence as a function of average daily bout cadence for each selected outcome by year. Results of Cox model with average daily steps, average daily bout cadence addition to other covariates: age, race, sex, coronary artery disease, cancer, body mass index, systolic blood pressure, education level, smoking, and alcohol use. Bout cadence referred to steps per minute computed by averaging the steps over the time when participant engaged in ≥2 consecutive minutes at ≥60 steps/minute across all the valid days. Shaded area represents 95%confidence interval.

Regardless of how step intensity was defined, that is, slow walking or moderate to vigorous activity, it was associated with lower risk of chronic diseases (all chunk tests *P* < 0.05, Supplementary Table 5 and Extended Data Fig. 10). The HR for step intensity for incident diabetes, hypertension, GERD, MDD, obesity and sleep apnea ranged from 0.43 to 0.88 (Supplementary Table 5). Step intensity (defined as slow walking) also remained significantly associated with obesity, sleep apnea, MDD, GERD and hypertension after adjusting for step count (all chunk tests *P* < 0.05). When defined using a moderate to vigorous intensity, bout cadence remained significantly associated with obesity, sleep apnea and GERD (all chunk tests *P* < 0.05; Supplementary Table 5).

## Discussion

We examined the association between step count volume and intensity across the entire spectrum of human disease using commercial activity monitors linked to an individual’s EHR. We identified consistent and statistically significant associations between activity levels and incident diabetes, hypertension, GERD, MDD, obesity and sleep apnea. Taking more steps each day was related to lower risk of developing these chronic diseases. Higher step counts were associated with protection from obesity in a high-risk population (BMI 25–29 kg m^–2^). Step count was positively correlated with step intensity, regardless of the bout cadence definition. The relation of step counts with disease risk persisted for diabetes, GERD, MDD and sleep apnea even when adjusting for step intensity. Step intensity was also significantly associated with these outcomes. These data provide new, empiric evidence of activity levels associated with chronic disease risk and suggest that integration of commercial wearables data into the EHR may be valuable to support clinical care.

Our findings are consistent with previous literature describing associations between step counts and adverse events^10,11^. A systematic review by Hall et al.^10^ found that taking more steps per day was related to lower risk of all-cause mortality, cardiovascular events and incident diabetes. The National Health and Nutrition Examination Survey study, which quantified steps over a 7-day monitoring period and assessed mortality over an average of 10.1 years, found a 51% lower mortality at 8,000 steps per day compared with 4,000 steps per day^1^. Similar results were reported from a middle-aged, biracial cohort with 7 days of monitoring and over 10 years of follow-up time^5^. A prospective cohort study conducted in 3,055 community-dwelling adults aged over 70 years found a similar nonlinear relation between daily steps and risk of developing diabetes, where the risk leveled off at 8,000 steps per day^12^. It is notable that step count thresholds associated with risk of mortality and cardiometabolic disease in prior studies are similar to step count thresholds associated with a wide variety of previously unreported phenotypes in our study. These results suggest that a single step count target of approximately 8,000–9,000 steps per day may be suitable to reduce risk of many common conditions.

Our study design and analytic approach differed from prior studies in important ways that make our results new and clinically relevant. First, prior studies assessed step counts over a single, short (usually 7 days) monitoring period with activity data between the baseline monitoring period and outcomes assessment, often many years later. Short monitoring periods are prone to an observer effect and may not accurately reflect true short- and long-term activity behavior^13^. In contrast, our models accounted for changes in steps over the entirety of an individual’s monitoring period (median of 4 years) rather than a brief snapshot. Second, prior studies have focused on a narrow set of outcomes (for example, mortality, diabetes and cardiovascular disease) ascertained at a single timepoint remote from the initial monitoring period. Our study used a hypothesis-generating phenome-wide association study approach, examining the association between step counts and the human phenome. In this manner, several new associations emerged including GERD, sleep apnea and MDD, which would likely go unidentified if disease phenotypes were selected a priori. Lastly, our analysis permitted incident disease to emerge at any point during clinical care rather than a prespecified follow-up time as performed in most cohort studies. One may speculate that this approach is more accurate with respect to the timing of incident disease and refines the temporal association between longitudinal activity and incident disease.

The findings of this study should be viewed in the context of several limitations. We were not able to account for daily step variations between different types of Fitbit models^14^ and seasonal differences^15^ as well as the occurrence of the COVID-19 pandemic because device data were not available at the time of analyses and data were date-shifted to protect privacy of participants. The characteristics of our study sample may limit the generalizability of our findings to more diverse populations. The majority of our cohort was relatively young, female, white and college-educated, and only included participants who owned Fitbit devices. Further, participants engaged in more steps per day (median 7,731 steps per day) than the average steps per day values reported for adults in the USA aged over 60 years^16^, suggesting that the analytical cohort in this study was more active. The fact that we were able to detect robust associations between steps and incident disease in this active sample suggests even stronger associations may exist in a more sedentary population. Therefore, further studies are needed including participants who are historically under-represented in biomedical research and those with activity levels that more closely mirror the general community.

Our data do not account for nonstepping activity such as swimming or cycling, such nonstepping movement is better captured via waveform or raw accelerometry and may provide additional insight into the association between physical activity and clinical diagnoses. Further, this study was observational in nature; therefore, causation should not be inferred. We acknowledge the potential for reverse causation in which the existence of a condition leads to taking fewer steps rather than the reverse. We attempted to mitigate this concern by focusing only on incident conditions and excluding any incident disease that emerged in the first 6 months of the monitoring period. Further, there is a potential for unmeasured confounding in our analyses because we were not able to account for an exhaustive list of potential confounders such as job status, environmental factors and differences in the usage patterns between participants over time^17^. Future studies are needed to investigate the impact of user behavior on health outcomes. Additionally, findings from exploratory logistic regression that did not find an association between steps per day and other outcomes such as cardiovascular diseases should be viewed with caution given that the analytical sample was relatively young, reported fewer outcomes and had limited follow-up. We excluded 15.4–16.0% (varies based on the outcome) of months due to fewer than 15 valid days of data in the Cox models. This missingness seems acceptable in comparison with prior studies which considered data to be valid if activity was captured on at least 3 out of 7 days (that is, up to 57% missing data)^18^. Lastly, we also acknowledge the limitations of using EHR data for outcomes ascertainment and the potential lack of specificity of diagnostic codes. It is possible that conditions are coded improperly, not coded at all or not recognized in the clinic. Nonetheless, our results reflect use of diagnostic codes in clinical practice across various medical systems, including large regional medical centers and federally qualified health centers.

Despite these limitations, the sources of data for our study are unique and offer an example of the potential clinical value of linking wearables data to the EHR. Published activity studies almost exclusively used research-grade actigraphs to measure steps and/or activity counts. In contrast, our data derive from commercially available devices. Although some fidelity is lost between research-grade and commercial devices, data from the latter are highly generalizable to a large portion of the public who own such devices. Activity data in this study date to the creation of a Fitbit account by the user. Therefore, the risk of an observer effect in this cohort is negligible because much of the activity data was collected before the participant consented to *All of Us*.

These findings may have important clinical and public health implications. We were unable to identify any published studies that investigated the association of physical activity data from a wearable device to health outcomes, defined using an individual’s EHR. Therefore, this study provides important new evidence that integration of these data sources is feasible and may provide valuable and actionable information for clinicians. Clinicians could monitor activity trends and provide evidence-based anticipatory guidance for activity tailored to an individual’s clinical characteristics and risk profile. For example, our data suggest that an individual with a BMI of 28 kg m^–2^ (can lower their risk of obesity 64% (95% CI 51, 80) by increasing steps from approximately 6,000 steps to 11,000 steps per day (Fig. 3). Although validation of these results is important, such data provide a necessary first step toward the development of personalized activity prescriptions. Further, wearables can also be used as an adjunct tool to encourage patients to engage in physical activity by allowing them to set, measure and track goals^19^. Finally, self-reported physical activity or exercise interventions may have potential beneficial effects to lower the incidence of depression^20^ and lower the severity of obstructive sleep apnea and associated comorbidities^21^. Therefore, these results provide support for the need for further research to examine the effect of real-world, unstructured physical activity to prevent or mitigate the effects of such conditions, including some previously unidentified activity-disease associations (for example, GERD).

In summary, using the data from AoURP, higher daily step counts were associated with reduced risk of several common, chronic diseases, including diabetes, hypertension, GERD, MDD, obesity and sleep apnea. This association between step counts over time and incident chronic diseases was consistent even after adjusting for potential covariates, including baseline steps per day and step intensity. Step intensity was also significantly associated with these incident diseases, although the relationships were less consistent than with step counts. These findings provide a new, robust source of evidence in support of the physical activity guidelines to prevent the risk of developing chronic diseases. If validated, these results may offer an evidence-base for refining activity recommendations based on an individual’s risk profile. This study also provides an example of the potential clinical value of linking data from commercially available wearables to the EHR.

## Methods

### Study participants

Participants aged over 18 years were enrolled after an informed consent process at clinics and regional medical centers that compose the AoURP network. A detailed description of AoURP has been published elsewhere^8^. For this study, we used the AoURP Registered Tier Dataset version 5 (R2021Q3R2 Curated Data Repository) available on the AoURP Researcher Workbench, a secure cloud-based platform. This dataset included information on physical measurements and vital signs collected at enrollment, surveys, EHR and Fitbit data from participants enrolled from May 30, 2018 to April 1, 2021. Our analyses focused on participants who owned a Fitbit and agreed to share their Fitbit and EHR data. We excluded participants who did not wear a Fitbit for at least 6 months. The *All of Us* Research Program Resource Access Board (RAB) has granted a post-hoc exception to the program’s Data and Statistics Dissemination Policy for reporting exact participant counts of less than 20 in some of the analyses reporting in this study, due to the very low risk to participant privacy and potential for re-identification.

### Fitbit data

Participants who provided primary consent to be part of the AoURP and share EHR data had an opportunity to provide their Fitbit data under the Bring Your Own Device program. Participants connected their own Fitbit device account with the AoURP Participant Portal and agreed to share their complete data over all time in their Fitbit account. For example, if a participant began tracking in their Fitbit account in May 2015 (that is, before the launch of AoURP), the AoURP data pull captured all existing Fitbit data in their account, not just recent data. A participant could stop sharing their data at any time. Participants’ data had direct identifiers removed and all datetime fields were subjected to date shifting by a random number between 1 and 365 days in accordance with approved AoURP privacy policies.

Fitbit data were reported as daily (steps per day) and intraday (steps per minute) step counts. We examined step intensity using steps per minute data^22,23^. Intensity was defined using mean bout cadence, that is, steps per minute, which were calculated by averaging the steps over the time when a participant engages in ≥2 consecutive minutes at ≥60 steps per minute (which suggests that the participant is at least engaged in slow walking^23^) across all valid days^1,23^. Evidence suggests that 10-hour wear time is sufficient to estimate daily physical activity during waking time^24^. Therefore, a valid day was defined as a participant wearing the Fitbit for at least 10 hours per day and reporting at least 100 steps per day. We acknowledge that Fitbit devices have reduced fidelity compared with research-grade actigraphs; however, in systematic reviews, Fitbits outperform other commercially available devices when correlated with research-grade devices^25,26^.

### Outcomes

The primary outcomes were identified using any incident billing code in EHR. We excluded any new diagnoses coded during the first 6 months of monitoring, assuming that such conditions were likely prevalent but not yet recognized clinically. The EHR data from different participating sites were mapped and harmonized using the Observational Medical Outcomes Partnership common data model^27–29^. We used the ICD to phecode map developed by Zheng et al.^30^ to map the EHR data to create phecodes (Supplementary Tables 1 and 2). We mapped ICD9CM and ICD10CM ‘source’ codes found in the AoURP Curated Data Repository to phecodes, which were used as outcomes.

### Statistical analyses

A CONSORT diagram was created to describe how many participants as well as Fitbit data, including percent days, were excluded based on the criteria used to create the analytical dataset. Descriptive statistics for participant’s demographic and clinical characteristics were presented by median and IQR for continuous variables and frequency for categorical variables. Mann–Whitney *U* and chi-squared tests for continuous and categorical variables, respectively, were used to compare these clinical characteristics for the participants that were excluded versus included in the analytical dataset for this study. We used logistic regression and Cox proportional hazard models to examine associations between step counts and incident disease. We first conducted multiple logistic regression models adjusted for age, sex and stated race, to examine the association between average steps per day over an individual’s entire monitoring period and all available phecodes. ORs and 95% CIs were reported per 1,000 step count increase. These analyses were exploratory in nature and allowed a data-driven approach to identify the diseases with a statistically significant relation with steps per day in a manner that was unconstrained by prior knowledge. The remainder of our analyses focused on disease associations by logistic regression that met a Bonferroni adjusted significance threshold and have a plausible biological link to physical activity. These conditions were then examined in separate continuous time-dependent Cox proportional hazard models with adjustment for relevant covariates. Participants were censored at their last medical encounter, which was defined as latest measurement, laboratory data, procedure or condition code.

The phecode definitions used to map the diseases that were used as an outcome for Cox model analyses can be found in Supplementary Table 1. Steps per day (averaged monthly) was examined as a repeated measure and time-varying variable to account for fluctuations in activity over an individual’s monitoring period. Only daily steps data before incident diagnoses were used in the Cox models. The time components for Cox models were chosen in terms of months. We also performed similar Cox model analyses restricted to individuals who were at high risk of incident obesity by virtue of a baseline BMI of 25–29.9 kg m^–2^. To examine whether the relationship between steps per day with the hazard of incident outcomes was linear or nonlinear, restricted cubic spline functions using 3, 4 and 5 knots of steps was fitted with separate Cox models. The model with lowest Akaike information criterion (AIC) value was chosen to then interpret the relation of steps per day with risk of developing a condition. Months for which participants had fewer than 15 days of observations were excluded from Cox models. We also examined the percent months and days that were excluded based on this additional criterion implemented in the Cox models.

To investigate the strength of association between step counts and risk of developing chronic disease, HRs and 95% CIs were computed by comparing 75th and 25th percentiles of daily step counts. We also conducted a falsification analysis to show that daily step counts did not associate diseases with no plausible relationship with activity; in this case, we tested carpal tunnel syndrome and actinic keratosis. All Cox models were adjusted for a priori covariates: age, sex (male, female), race (Black or African American, white, other), coronary artery disease (CAD) (yes, no), cancer (yes, no), BMI, systolic blood pressure, education level (no college, some college, college degree), all time smoking (<100 cigarettes, ≥100 cigarettes) and alcohol use (alcohol participant, not an alcohol participant). All covariates except BMI, systolic blood pressure, CAD and cancer were assessed at enrollment visit via participant surveys. Baseline BMI and systolic blood pressure was extracted using EHR data. CAD and cancer were ascertained using ICD9CM/ICD10CM or Current Procedural Terminology (CPT4) codes as well as ICD9CM/ICD10CM codes, respectively. These codes to define CAD and cancer are shown in Supplementary Table 1.

In addition to accounting for a priori covariates, we ran a separate Cox model accounting for wear time, which was considered to be a time-varying covariate. Specifically, wear time was defined as the number of hours in a day that contained non-zero step counts. We also performed trajectory analyses by examining the average daily step counts over 0–3 months, 3–6 months, 6–12 months and 12–24 months for the participants who developed versus those who did not develop the conditions, which were examined in the Cox models. We then accounted for baseline daily steps averaged over the first 3 and 6 months, in separate Cox models in addition to a priori covariates, in an attempt to mitigate the potential for reverse causation.

To examine the relation between steps per day and step intensity (bout cadence), the Spearman correlation coefficient was computed. Additionally, we descriptively examined the gradient of disease risk by plotting the intersections of daily step counts and bout cadence quartiles. We also used the probability density plot to examine the association between daily step counts and bout cadence on a continuous spectrum for participants who developed versus those who did not develop the conditions, which were examined in Cox models. We conducted similar Cox analyses to investigate whether the association of steps per day with the risk of developing chronic conditions stayed consistent after accounting for step intensity and potential covariates. Similarly, we used a Cox model adjusted for a priori covariates to examine the strength of association between step intensity and outcomes. Lastly, we repeated these analyses, using step intensity, which referred to steps per minute computed by averaging the steps over the time when a participant engaged in ≥2 consecutive minutes at ≥100 steps per minute, a threshold used to determine time spent in moderate to vigorous activity^23^, across all the valid days.

Proportional hazards assumption was examined using cox.zph R function^31^ in the survival R package. Proportional hazard assumptions were met for all models. All missing data for covariates were imputed using multiple imputation with predictive mean matching^32^. The rms package^33^ was used to fit all Cox models and to compute HRs. The ‘anova’ function in the rms package was used to assess whether the predictors were significantly associated with the outcome as well as to evaluate significance of nonlinear effects for steps based on the model with the lowest AIC value. Specifically, we performed a Wald *χ*^2^ test (or ‘chunk test’) to jointly assess whether all the terms, including nonlinear terms in the restricted cubic spline are zero^34^. If the test is nonsignificant, it indicates that the variable represented by the spline is not associated with the outcome or it does not have a nonlinear relationship with the outcome. The aregImpute function in the Hmisc R package^35^ was used to conduct multiple imputation and all the results were pooled across the five imputation datasets.

### Reporting summary

Further information on research design is available in the Nature Research Reporting Summary linked to this article.

## Online content

Any methods, additional references, Nature Research reporting summaries, source data, extended data, supplementary information, acknowledgments, peer review information; details of author contributions and competing interests; and statements of data and code availability are available at 10.1038/s41591-022-02012-w.

## Supplementary information

Supplementary Information Supplementary Tables 1–5.

Reporting Summary

## Data Availability

To ensure privacy of participants, data used for this study are available to approved researchers following registration, completion of ethics training and attestation of a data use agreement through the *All of Us* Research Workbench platform, which can be accessed via https://workbench.researchallofus.org/login.

## Code availability

Code used for this study can be made available to users of the *All of Us* Research Workbench platform by contacting our study team.
