# Author Correction: Association of step counts over time with the risk of chronic disease in the *All of Us* Research Program

**DOI:** 10.1038/s41591-023-02313-8

**Published:** 2023-04-12

**Authors:** Hiral Master, Jeffrey Annis, Shi Huang, Joshua A. Beckman, Francis Ratsimbazafy, Kayla Marginean, Robert Carroll, Karthik Natarajan, Frank E. Harrell, Dan M. Roden, Paul Harris, Evan L. Brittain

**Affiliations:** 1 Vanderbilt Institute of Clinical and Translational Research, Vanderbilt University Medical Center, Nashville, TN USA; 2 Department of Biostatistics, Vanderbilt University School of Medicine, Nashville, TN USA; 3 Division of Cardiovascular Medicine, Vanderbilt University Medical Center, Nashville, TN USA; 4 Department of Biomedical Informatics, Vanderbilt University Medical Center, Nashville, TN USA; 5 Department Biomedical Informatics, Columbia University, New York, NY USA; 6 Department of Medicine and Biomedical Informatics, Vanderbilt University Medical Center, Nashville, TN USA; 7 Department of Biomedical Informatics, Biomedical Engineering and Biostatistics, Vanderbilt University Medical Center, Nashville, TN USA

**Keywords:** Risk factors, Preventive medicine

Correction to: *Nature Medicine* 10.1038/s41591-022-02012-w. Published online 10 October 2022.

In the version of this article initially published, the statement now reading “The *All of Us* Research Program Resource Access Board (RAB) has granted a post-hoc exception to the program’s Data and Statistics Dissemination Policy for reporting exact participant counts of less than 20 in some of the analyses reporting in this study, due to the very low risk to participant privacy and potential for re-identification” was mistakenly omitted from the “Study participants” subsection within the Methods. The error has been corrected in the HTML and PDF versions of the article.

