# Supplementary material for: Association of step counts over time with the risk of chronic disease in the All of Us Research Program
Source: Nat Med. 2022 Oct 10;28(11):2301–8. doi: 10.1038/s41591-022-02012-w (PMC9671804; doi:10.1038/s41591-022-02012-w)
Supplement: Supplementary file 1 — Supplementary Tables 1–5. [file 41591_2022_2012_MOESM1_ESM.pdf]

nature medicine

Article https://doi.org/10.1038/s41591-022-02012-w

Association of step counts over time with the risk of chronic disease in the *All of Us* Research Program

In the format provided by the authors and unedited

Supplemental Materials

Supplementary Tables 2

Supplementary Table 1. Definitions used to map EHR data to conditions across human phenome and these definitions are used to define outcomes and covariates in Cox models 2

Supplementary Table 2. Top 50 associated phecodes from adjusted logistic regression by p-value. 3

Supplementary Table 3. Hazard ratios and 95% confidence intervals for obesity comparing 75th percentile step count (10,900), vs 25th percentile step count (6,500) specifing BMI at a given value between 25-29 kg/m^2. 4

Supplementary Table 4. Hazard ratios and 95% confidence intervals comparing 75th percentile, vs 25th percentile step counts. 4

Supplementary Table 5. Hazard ratios and 95% confidence intervals comparing 75th percentile, vs 25th percentile bout cadence (step intensity). 5

Supplementary Tables

Supplementary Table 1. Definitions used to map EHR data to conditions across human phenome and these definitions are used to define outcomes and covariates in Cox models

Definition Name	EHR data
Obesity	Phecode: 278.1
Diabetes	Phecode: 250.2, 250.24
Sleep Apnea	Phecode: 327.3, 327.32
Major depressive disorder	Phecode: 296.22
GERD	Phecode: 530.11
Essential hypertension	Phecode: 401.1
Coronary Artery Disease (CAD)	ICD9/10: 410,410.*,411,411.*,412,412.*,413,413.*,414,414.*,V45.82 I25.1*<br>CPT: 33534, 33535, 33536, 33510, 33511, 33512, 33513, 33514, 33515, 33516, 33517, 33518, 33519, 33520, 33521, 33522, 33523, 92980, 92981, 92982, 92984, 92995, 92996
Cancer	ICD9/10: 104, 104.*, 105, 105.*, 106, 106.*…<br>207, 207.*, 208, 208.*, 209, 209.*<br>C00, C00.*, C01, C01.*, …<br>D47, D47.*, D48, D48.*, D49, D49.*

Supplementary Table 2. Top 50 associated phecodes from adjusted logistic regression by p-value.

OR is odds ratio per 1000 step increase. N is sample size. 2.5% and 97.5% are lower and upper 95% CI bounds, respectively. Asterisks represent significance at Bonferroni corrected alpha level of 3.1856×10^{-5}.

Phecode	Name	N	Events	Odds Ratio	2.5%	97.5%	p-value
327.32	Obstructive sleep apnea	5518	342	0.88	0.84	0.92	<0.0001 *
278.1	Obesity	5267	380	0.89	0.86	0.93	<0.0001 *
327.3	Sleep apnea	5699	183	0.85	0.80	0.90	<0.0001 *
357	Inflammatory and toxic neuropathy	5923	139	0.84	0.78	0.89	<0.0001 *
250.24	Type 2 diabetes with neurological manifestations	5976	37	0.69	0.60	0.79	<0.0001 *
345.3	Convulsions	5960	26	0.62	0.51	0.75	<0.0001 *
585.1	Acute renal failure	5973	71	0.79	0.72	0.87	<0.0001 *
401.1	Essential hypertension	4897	498	0.92	0.89	0.95	<0.0001 *
296.22	Major depressive disorder	5370	483	0.92	0.89	0.95	<0.0001 *
530.11	GERD	5091	451	0.92	0.89	0.95	<0.0001 *
428.4	Heart failure with preserved EF [Diastolic heart failure]	6020	33	0.71	0.61	0.82	<0.0001 *
789	Nausea and vomiting	5488	309	0.91	0.87	0.95	<0.0001 *
599.4	Urinary incontinence	5815	127	0.86	0.81	0.92	<0.0001 *
512.7	Shortness of breath	5493	379	0.92	0.89	0.95	<0.0001 *
851	Complications of transplants and reattached limbs	5990	57	0.79	0.71	0.88	<0.0001 *
276.14	Hypopotassemia	5863	107	0.85	0.79	0.92	<0.0001 *
532	Dysphagia	5777	166	0.89	0.84	0.94	<0.0001 *
770	Myalgia and myositis unspecified	5575	145	0.88	0.82	0.93	<0.0001
274.1	Gout	5975	39	0.77	0.68	0.87	<0.0001
427.7	Tachycardia NOS	5863	145	0.88	0.83	0.93	<0.0001
418	Nonspecific chest pain	5131	381	0.93	0.89	0.96	<0.0001
338.1	Acute pain	5803	302	0.92	0.88	0.96	<0.0001
512.9	Other dyspnea	5472	378	0.93	0.90	0.96	<0.0001
428.1	Congestive heart failure (CHF) NOS	5979	31	0.75	0.65	0.87	<0.0001
475	Chronic sinusitis	5495	194	0.90	0.86	0.95	0.0001
785	Abdominal pain	4833	545	0.94	0.91	0.97	0.0001
599.3	Dysuria	5582	255	0.92	0.88	0.96	0.0001
355.1	Chronic pain syndrome	5979	52	0.81	0.73	0.90	0.0002
480	Pneumonia	5804	94	0.87	0.80	0.93	0.0002
450	Noninfectious disorders of lymphatic channels	6014	29	0.74	0.64	0.87	0.0002
250.2	Type 2 diabetes	5634	163	0.90	0.85	0.95	0.0002
571.5	Other chronic nonalcoholic liver disease	5868	164	0.90	0.85	0.95	0.0003
782.3	Edema	5749	215	0.91	0.87	0.96	0.0003
386.9	Dizziness and giddiness (Light-headedness and vertigo)	5475	337	0.93	0.90	0.97	0.0003
715	Other inflammatory spondylopathies	6016	28	0.76	0.65	0.88	0.0004
292	Neurological disorders	5971	77	0.86	0.79	0.93	0.0004
41.9	Infection with drug-resistant microorganisms	6031	8	0.49	0.33	0.73	0.0004
288.2	Elevated white blood cell count	5936	85	0.87	0.80	0.94	0.0005
741.3	Difficulty in walking	6006	52	0.83	0.75	0.92	0.0005
338.2	Chronic pain	5515	638	0.95	0.92	0.98	0.0005
495	Asthma	5429	266	0.92	0.88	0.97	0.0005
296.1	Bipolar	5925	47	0.81	0.72	0.91	0.0005
428.3	Heart failure with reduced EF [Systolic or combined heart failure]	6012	30	0.78	0.68	0.90	0.0005
340	Migraine	5464	221	0.92	0.87	0.96	0.0006
550.2	Diaphragmatic hernia	5861	140	0.90	0.85	0.96	0.0007
276.41	Acidosis	6008	32	0.79	0.68	0.90	0.0007
401.21	Hypertensive heart disease	5995	39	0.81	0.72	0.92	0.0007
327.41	Organic or persistent insomnia	5965	81	0.87	0.80	0.94	0.0008
350.1	Abnormal involuntary movements	5895	143	0.90	0.85	0.96	0.0008
596.5	Functional disorders of bladder	5986	49	0.83	0.75	0.93	0.0008

Supplementary Table 3. Hazard ratios and 95% confidence intervals for obesity comparing 75th percentile step count (10,900), vs 25th percentile step count (6,500) specifing BMI at a given value between 25-29 kg/m^2.

BMI	*HR	95% CI	*p*-value
25	0.25	0.13-0.49	<0.001
26	0.34	0.21-0.56	<0.001
27	0.47	0.33-0.65	<0.001
28	0.64	0.51-0.8	<0.001
29	0.87	0.67-1.14	0.312

*adjusted for age, race, sex, coronary artery disease, cancer, systolic blood pressure, education level, smoking, alcohol use, body mass index, and body mass index*steps counts interaction

Supplementary Table 4. Hazard ratios and 95% confidence intervals comparing 75th percentile, vs 25th percentile step counts.

Model/Diagnosis	25th Percentile (thous.)	75th Percentile (thous.)	HR (75% vs 25%)	95% CI	*p*-value
**Model 1 w/ bout cadence (≥60 steps/minute)**					
Diabetes	6.37	10.79	0.75	0.57-0.97	0.029
Hypertension	6.49	10.88	0.84	0.64-1.10	0.195
GERD	6.45	10.92	0.81	0.69-0.94	0.007
MDD	6.39	10.88	0.74	0.63-0.87	<0.001
Obesity	6.46	10.92	0.67	0.47-0.94	0.019
Sleep Apnea	6.43	10.85	0.64	0.53-0.77	<0.001
**Model 2 w/ bout cadence (≥100 steps/minute)**					
Diabetes	8.63	12.60	0.76	0.61-0.96	0.019
Hypertension	8.65	12.60	0.83	0.72-0.95	0.007
GERD	8.67	12.68	0.86	0.74-1.01	0.058
MDD	8.64	12.65	0.84	0.73-0.98	0.026
Obesity	8.67	12.66	0.80	0.56-1.14	0.218
Sleep Apnea	8.64	12.61	0.73	0.62-0.87	<0.001

Results of Cox model with average daily steps, average daily bout cadence addition to other covariates: age, race, sex, coronary artery disease, cancer, body mass index, systolic blood pressure, education level, smoking, and alcohol use. Model 1 with bout cadence (≥60 steps/minute) consists of bout cadence, which referred to steps per minute computed by averaging the steps over the time when participant engaged in ≥2 consecutive minutes at ≥60 steps/minute across all the valid days. Model 2 with bout cadence (≥100 steps/minute) consists of bout cadence, which referred to steps per minute computed by averaging the steps over the time when participant engaged in ≥2 consecutive minutes at ≥100 steps/minute across all the valid days.

Supplementary Table 5. Hazard ratios and 95% confidence intervals comparing 75th percentile, vs 25th percentile bout cadence (step intensity).

Model/Diagnosis	25th Percentile (steps/min)	75th Percentile (steps/min)	HR (75% vs 25%)	95% CI	*p*-value
**Model 1 w/ bout cadence (≥60 steps/minute), no daily steps**					
Diabetes	83.62	96.15	0.78	0.62-0.98	0.032
Hypertension	84.09	96.58	0.71	0.62-0.81	<0.001
GERD	83.86	96.39	0.66	0.57-0.76	<0.001
MDD	83.67	96.28	0.66	0.58-0.76	<0.001
Obesity	83.86	96.50	0.65	0.48-0.89	0.007
Sleep Apnea	83.84	96.38	0.43	0.32-0.59	<0.001
**Model 2 w/ bout cadence (≥60 steps/minute) and daily steps**					
Diabetes	84.18	96.55	0.88	0.68-1.15	0.348
Hypertension	84.64	96.95	0.89	0.68-1.17	0.393
GERD	84.42	96.78	0.72	0.61-0.85	<0.001
MDD	84.22	96.68	0.78	0.67-0.92	0.002
Obesity	84.40	96.87	0.70	0.51-0.96	0.026
Sleep Apnea	84.40	96.77	0.72	0.60-0.85	<0.001
**Model 3 w/ bout cadence (≥100 steps/minute), no daily steps**					
Diabetes	109.41	117.00	0.72	0.56-0.92	0.010
Hypertension	109.54	117.20	0.88	0.78-1.00	0.050
GERD	109.45	117.13	0.80	0.69-0.92	0.002
MDD	109.39	117.04	0.86	0.76-0.98	0.027
Obesity	109.47	117.14	0.70	0.51-0.97	0.032
Sleep Apnea	109.45	117.09	0.80	0.67-0.95	0.010
**Model 4 w/ bout cadence (≥100 steps/minute) and daily steps**					
Diabetes	109.41	117.00	0.76	0.58-0.98	0.037
Hypertension	109.54	117.20	0.92	0.81-1.05	0.230
GERD	109.45	117.13	0.83	0.71-0.96	0.012
MDD	109.39	117.04	0.90	0.78-1.03	0.118
Obesity	109.47	117.14	0.73	0.53-1.02	0.061
Sleep Apnea	109.45	117.09	0.85	0.72-1.02	0.081

Results of Cox model with covariates: age, race, sex, coronary artery disease, cancer, body mass index, systolic blood pressure, education level, smoking, and alcohol use.
Model 1 w/ bout cadence (≥60 steps/minute), no daily steps = Covarites + bout cadence, which referred to steps per minute computed by averaging the steps over the time when participant engaged in ≥2 consecutive minutes at ≥60 steps/minute across all the valid days.
Model 2 w/ bout cadence (≥60 steps/minute) and daily steps = Model 1 +average daily steps
Model 3 w/ bout cadence (≥ 100 steps/minute), no daily steps = Model 1, where bout cadence is computed by averaging the steps over the time when participant engaged in ≥2 consecutive minutes at

≥100 steps/minute across all the valid days.
Model 4 w/ bout cadence (≥ 100 steps/minutes), w/ daily steps = Model 3 + average daily steps
