# Supplementary material for: Association of step counts over time with the risk of chronic disease in the All of Us Research Program
Source: Nat Med. 2022 Oct 10;28(11):2301–8. doi: 10.1038/s41591-022-02012-w (PMC9671804; doi:10.1038/s41591-022-02012-w)
Supplement: Supplementary file 2 — Reporting Summary [file 41591_2022_2012_MOESM2_ESM.pdf]

nature portfolio

Corresponding author(s): Evan Brittain

Last updated by author(s): Aug 2, 2022

Reporting Summary

Nature Portfolio wishes to improve the reproducibility of the work that we publish. This form provides structure for consistency and transparency in reporting. For further information on Nature Portfolio policies, see our Editorial Policies and the Editorial Policy Checklist.

Statistics

For all statistical analyses, confirm that the following items are present in the figure legend, table legend, main text, or Methods section.

n/a	Confirmed	
☐	☒	The exact sample size (*n*) for each experimental group/condition, given as a discrete number and unit of measurement
☐	☒	A statement on whether measurements were taken from distinct samples or whether the same sample was measured repeatedly
☐	☒	The statistical test(s) used AND whether they are one- or two-sided<br>*Only common tests should be described solely by name; describe more complex techniques in the Methods section.*
☐	☒	A description of all covariates tested
☐	☒	A description of any assumptions or corrections, such as tests of normality and adjustment for multiple comparisons
☐	☒	A full description of the statistical parameters including central tendency (e.g. means) or other basic estimates (e.g. regression coefficient) AND variation (e.g. standard deviation) or associated estimates of uncertainty (e.g. confidence intervals)
☐	☒	For null hypothesis testing, the test statistic (e.g. *F*, *t*, *r*) with confidence intervals, effect sizes, degrees of freedom and *P* value noted<br>*Give P values as exact values whenever suitable.*
☒	☐	For Bayesian analysis, information on the choice of priors and Markov chain Monte Carlo settings
☐	☒	For hierarchical and complex designs, identification of the appropriate level for tests and full reporting of outcomes
☐	☒	Estimates of effect sizes (e.g. Cohen's *d*, Pearson's *r*), indicating how they were calculated

Our web collection on statistics for biologists contains articles on many of the points above.

Software and code

Policy information about availability of computer code

Data collection	Participants aged over 18 years old were enrolled after an informed consent process at clinics and regional medical centers that compose the All of Us Research Program network.
Data analysis	To ensure participant's privacy, data from All of Us Research program can be accessed and analyzed through a secured cloud-based platform - Researcher Workbench. Specifically, we used R programming language within Jupyter notebook to conduct analysis. Code used for this study can be made available to users of the All of Us Research Workbench platform by contacting our study team.

For manuscripts utilizing custom algorithms or software that are central to the research but not yet described in published literature, software must be made available to editors and reviewers. We strongly encourage code deposition in a community repository (e.g. GitHub). See the Nature Portfolio guidelines for submitting code & software for further information.

Data

Policy information about availability of data

All manuscripts must include a data availability statement. This statement should provide the following information, where applicable:

- Accession codes, unique identifiers, or web links for publicly available datasets
- A description of any restrictions on data availability
- For clinical datasets or third party data, please ensure that the statement adheres to our policy

Data used for this study is available to approved researchers following registration, completion of ethics training, and attestation of a data use agreement through the All of Us Research Workbench platform, which can be accessed via https://workbench.researchallofus.org/login.

Human research participants

Policy information about studies involving human research participants and Sex and Gender in Research.

Reporting on sex and gender	In this study, we have used sex assigned at birth to define the participant characteristics as well as a covariate in the analytical models.
Population characteristics	Participants had a median age of 56.7 years IQR[41.5, 67.6] and median BMI of 28.1 kg/m2 IQR[24.3, 32.9] at baseline. Nearly 73%, 84% and 71% were female, White, and with a college degree, respectively.
Recruitment	Participants aged over 18 years old were enrolled after an informed consent process at clinics and regional medical centers that compose the All of Us Research Program network.
Ethics oversight	The All of Us Research Program uses high-quality security technology to help keep participant data safe. The data that participants give to the All of Us Research Program are personal. To protect this information, the program follows strict security protocols and processes that are regularly reviewed and updated by the Institutional Review Board (IRB) of the All of Us Research Program.<br><br>Further, the row-level participant's data can only accessed by registered users on the All of us Researcher Workbench - secured cloud based platform. Further, all the registered users have successfully completed the mandatory education training on conducting responsible and ethical research with data from All of Us participants

Note that full information on the approval of the study protocol must also be provided in the manuscript.

Field-specific reporting

Please select the one below that is the best fit for your research. If you are not sure, read the appropriate sections before making your selection.

☐ Life sciences ☒ Behavioural & social sciences ☐ Ecological, evolutionary & environmental sciences

For a reference copy of the document with all sections, see nature.com/documents/nr-reporting-summary-flat.pdf

Behavioural & social sciences study design

All studies must disclose on these points even when the disclosure is negative.

Study description	Observational longitudinal cohort study.
Research sample	6042 participants who possess their own Fitbit and consented to link activity data and electronic health records (EHR) within the All of Us Research Program and [illegible]6 months of Fitbit monitoring data.
Sampling strategy	All of Us Research Program is an initiative that is accumulating multiple streams of health-related information (e.g., electronic health records (EHRs), genomics, physical measures, participant surveys and wearables such as Fitbit) in 1,000,000 or more Americans and includes a focus on populations usually under-represented in biomedical research to date.
Data collection	For this study, we used the All of Us Registered Tier Dataset version 5 (R2021Q3R2 Curated Data Repository) available on the All of Us Researcher Workbench, a secure cloud-based platform. This dataset included information on physical measurements and vital signs collected at enrollment, surveys, EHR and Fitbit data from participants enrolled from May 30, 2018 to April 1, 2021. Our analyses focused on participants who owned a Fitbit and agreed to share their Fitbit and EHR data. We excluded participants who did not wear a Fitbit for at least 6 months.
Timing	For this study, data from participants enrolled from May 30, 2018 to April 1, 2021 in All of Us Research Program were used.
Data exclusions	Of the 329,070 All of Us participants available at the time of our analysis, 214,206 participants had consented to share EHR data. Of

Data exclusions	those sharing EHR data, 6,042 participants linked their own Fitbit device, had valid Fitbit data over at least 6 months of total monitoring and were aged at least 18 years at any time during the monitoring period.
Non-participation	We are using data from All of Us Research Program. As noted in the All of Us Responsible Conduct of Research training, the Researcher Workbench employs a data passport model, through which we are not conducting human subjects research with All of Us data for two reasons:(1) The research will not directly involve participants, however, it only includes their data; and (2) the data available in the Researcher Workbench has been carefully checked and altered to remove identifying information while preserving its scientific utility. Therefore, we do not have access to information around non-participation to the program. However, for our study, of the 329,070 All of Us participants available at the time of our analysis, 323,028 participants were not included in the analyses because they did not consent to share EHR data or had missing/invalid Fitbit data.
Randomization	Observational longitudinal study design was employed to investigate the research question. Therefore, instead of randomization, covariate adjustment method was used. All Cox models were adjusted for age, sex (Male, Female), race (Black or African American, White, other), coronary artery disease [CAD] (yes, no), cancer (yes, no), body mass index (BMI), systolic blood pressure, Education level (no college, some college, college degree), all time smoking (< 100 cigarettes, >= 100 cigarettes), and alcohol use (alcohol participant, not an alcohol participant). All covariates except BMI, systolic blood pressure, CAD and cancer were assessed at enrollment visit via participant surveys. Baseline BMI and systolic blood pressure was extracted using EHR data. CAD and cancer were ascertained using ICD9CM/ICD10CM or Current Procedural Terminology (CPT4) codes as well as ICD9CM/ICD10CM codes, respectively.

Reporting for specific materials, systems and methods

We require information from authors about some types of materials, experimental systems and methods used in many studies. Here, indicate whether each material, system or method listed is relevant to your study. If you are not sure if a list item applies to your research, read the appropriate section before selecting a response.

Materials & experimental systems

n/a	Involved in the study
☒	☐ Antibodies
☒	☐ Eukaryotic cell lines
☒	☐ Palaeontology and archaeology
☒	☐ Animals and other organisms
☒	☐ Clinical data
☒	☐ Dual use research of concern

Methods

n/a	Involved in the study
☒	☐ ChIP-seq
☒	☐ Flow cytometry
☒	☐ MRI-based neuroimaging
